# Aberrant *Ascaris suum* Nematode Infection in Cattle, Missouri, USA

**DOI:** 10.3201/eid2202.150686

**Published:** 2016-02

**Authors:** Holly L. Taylor, Sean T. Spagnoli, Michael J. Calcutt, Dae Young Kim

**Affiliations:** University of Missouri, Columbia, Missouri, USA (H.L. Taylor, M.J. Calcutt, D.Y. Kim);; Oregon State University, Corvallis, Oregon, USA (S.T. Spagnoli)

**Keywords:** Ascaris suum, nematode, cattle, aberrant infection, outbreak, Missouri, United States

**To the Editor:** *Ascarididae* is a family of parasitic nematodes, commonly known as intestinal roundworms, that affects humans and various animals, including pigs, dogs, cats, horses, raccoons, and marine mammals. The *Ascaris suum* nematode is a common parasite of swine. There has been a sizeable decrease in the number of cases of infection with this nematode because swine husbandry has become more modernized and industrialized (*1*).

The *A. lumbricoides* nematode is the primary roundworm of humans. However, sporadic aberrant *A. suum* nematode infections have been reported in humans worldwide. Although uncommon in industrialized countries, several human cases of infection with this nematode have been reported (*2**,**3*), including an outbreak in Maine, USA, in 2010–2013 (*4*). Similar to human cases, cases of aberrant infection of *A. suum* nematodes in cattle are rare; no infections have been reported in North America since the 1960s (*5*).

In September 2010, a 1.5-year-old heifer from a farm was brought to Veterinary Medical Diagnostic Laboratory (VMDL), University of Missouri (Columbia, MO, USA), for postmortem examination. The heifer died with clinical signs of respiratory distress, coughing, tachypnea, and general weakness. The farm contained 15 heifers, all of which had shown similar clinical signs for 2–3 weeks. Two heifers had died 2 days before being brought to the VMDL but no necropsy was performed. New cattle had not recently been introduced to the herd, and animals were current for all vaccinations.

Necropsy showed bilaterally inflated lungs with moderate emphysema, diffusely firm red parenchyma, and abundant blood-tinged froth in the bronchi and trachea. Interstitial pneumonia was diagnosed on the basis of gross appearance, and viral pneumonia or acute bovine pulmonary emphysema and edema, especially that caused by toxic Perilla mint (*Peri indicutescens*), was suspected. However, microscopic examination showed diffuse, severe, fibrinous, eosinophilic and histiocytic interstitial pneumonia and multiple nematode larvae in bronchi and alveolar sacs (Figure). Nematode larvae were ≈100 µm in diameter and had a cuticle, pseudocoelom, coelomyarian musculature, large lateral cords, lateral alae, and a digestive tract. Bacteriological and molecular diagnostic tests did not indicate a viral or bacterial etiology. Fecal examination showed few coccidian and strongylid eggs.

**Figure:**
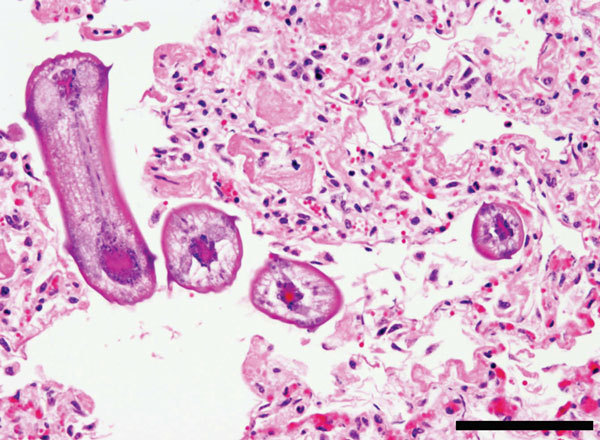
Multiple cross-section of *Ascaris suum* nematode larvae in the lung of cattle. Larvae have prominent lateral alae and lateral cords. Several scattered eosinophils and macrophages and abundant fibrin are also shown. Scale bar indicates 200 µm.

*Dictyocaulus viviparous,* a trichostrongyle, is the pathogenic bovine lungworm. However, morphologic characteristics of nematodes in this study were more consistent with ascarids. There is a bovine roundworm (*Toxocara viturolum*), but it is uncommon in the United States. In tropical and subtropical regions, especially in Africa and Southeast Asia, *T. viturolum* roundworms infect suckling bovine calves (*6*). *T. viturolum* roundworms have been identified in Florida, USA, in 2010 (*7*) and Canada in 2012 (*8*). Although *T. viturolum* roundworms cause diarrhea and weight loss in calves <3 months of age, infection with these roundworms is largely asymptomatic in adults.

Further investigation indicated that the source of infection was a pair of young-adult, free-range pigs living alongside the affected cattle. The cattle had been fed a round bale of hay to which the pigs had access. Parasitologic examination of the hay bale showed contamination with *A. suum* nematode eggs, indicating that the hay bale was probably exposed to pig feces. The hay bale was removed from the remaining cattle, and clinical signs gradually resolved without additional loss.

To unambiguously identify the nematodes, genomic DNA was extracted from formalin-fixed, paraffin-embedded tissue of infected lung. On the basis of the mitochondrial DNA sequence of *A. suum*, primers were designed to amplify a partial sequence encoding NADH dehydrogenase subunit 5: VMDL-F2 (5′-TGCTAAAGGTTGGGTTTATGGA-3′) and M3-R (5′-CCTACTGCGTAGAGCCAGA-3′). PCR was performed by using the GoTaq Hot Start Green Master Mix (Promega, Madison, WI, USA) under the following conditions: 95°C for 4 min; 45 cycles of 94°C for 45 sec, 52°C for 45 sec, and 72°C for 1 min; and final extension at 72°C for 5 min.

The resulting amplicon was purified by using spin chromatography (QIAGEN, Valencia, CA, USA) and sequenced with amplification primers at the University of Missouri DNA Core Facility. The resulting 354-bp sequence (GenBank accession no. KT808321) was compared with sequences in GenBank by using blastn (https://blast.ncbi.nlm.nih.gov/Blast.cgi?PAGE_TYPE=BlastSearch) and found to be 100% identical with that of *A. suum* nematode, thus confirming the identity. The sequence was only 98.3% identical with that of *A. lumbricoides* nematode, the species that had the next closest match (6 polymorphisms).

Increasing interest in and demand for organic meat results in proliferation of small, suburban farms that raise free-range animals. Reports have warned the community about major increases in zoonotic parasitic infections in organically raised pigs compared with animals raised under modern husbandry practices (*9**,**10*). The recent zoonotic case in Maine also involved a farm that grew and sold organic vegetables and organic livestock, including pigs (*4*). Until new proven preventive protocols are established, health personnel and veterinarians should be well informed about the risk for aberrant parasitic infections in pigs and possible transmission to humans and other domestic animals.
